# Metabolic Dysfunction-Associated Steatotic Liver Disease (MASLD): the interplay of gut microbiome, insulin resistance, and diabetes

**DOI:** 10.3389/fmed.2025.1618275

**Published:** 2025-08-14

**Authors:** Ankita Dua, Rashmi Kumari, Mona Singh, Roushan Kumar, Sunila Pradeep, Akinyemi I. Ojesina, Roshan Kumar

**Affiliations:** ^1^Department of Zoology, Shivaji College, University of Delhi, New Delhi, India; ^2^Department of Zoology, College of Commerce, Arts & Science, Patliputra University, Patna, Bihar, India; ^3^Department of Obstetrics and Gynecology, Division of Gynecologic Oncology, Medical College of Wisconsin, Milwaukee, WI, United States; ^4^Post-Graduate Department of Zoology, Magadh University, Bodh Gaya, Bihar, India; ^5^Medical College of Wisconsin Cancer Center, Milwaukee, WI, United States; ^6^Department of Microbiology and Immunology, Medical College of Wisconsin, Milwaukee, WI, United States

**Keywords:** MASLD, MASH, NAFLD, NASH, microbiome, dysbiosis, metabolic syndrome

## Abstract

The global prevalence of Metabolic Dysfunction-Associated Steatotic Liver Disease (MASLD) has reached alarming levels, affecting nearly one-third of the world's population. This review analyzes current evidence on the intricate relationships between MASLD, insulin resistance, and type 2 diabetes mellitus (T2DM), with particular emphasis on gut microbiome interactions. As MASLD progresses from simple steatosis to Metabolic Dysfunction-Associated Steatohepatitis (MASH), it can lead to severe complications including fibrosis, cirrhosis, and hepatocellular carcinoma. The pathogenesis of MASLD is multifactorial, involving hepatic lipid accumulation, oxidative stress, inflammation, and dysregulation of the gut-liver axis. Insulin resistance is a central driver of disease progression, closely linked to obesity and metabolic syndrome. Recent research highlights how gut microbiome dysbiosis exacerbates MASLD through mechanisms such as increased intestinal permeability, systemic inflammation, and altered metabolic signaling. Identification of microbial signatures offers promise for novel diagnostic and therapeutic strategies. By integrating metabolic, inflammatory, and microbial perspectives, this review provides a comprehensive overview of MASLD pathogenesis and its association with obesity, insulin resistance, and T2DM.

## Introduction

MASLD has emerged as the most common liver disease worldwide and has become a major health burden in both developed and emerging countries (1, 2). Its global prevalence is high, affecting roughly 30% of the population, and has shown an alarming 50.4% relative increase between 1990 and 2019 (3). MASLD is a disorder characterized by hepatic steatosis [fat deposition in > 5% hepatocytes (4)]; when no other cause for secondary fat accumulation like excess alcohol consumption can be identified. It can be diagnosed in a patient meeting one out of five cardiovascular risk factors (5). It ranges from benign non-inflammatory condition (NAFL) to severe MASLD which includes portal and lobular inflammation (6). Without intervention, MASLD may progress to fibrosis, cirrhosis, or even hepatocellular carcinoma (HCC) (7, 8). MASLD is the hepatic manifestation of metabolic syndrome (metS), driven by genetic variants like PNPLA3 rs738409 G and strongly associated with metabolic comorbidities, including obesity, T2DM, hyperlipidaemia and hypertension (8–10). MASLD is now recognized as a multifactorial disease, and recent literature proposes this renaming to better reflect its metabolic origins and remove alcohol-related exclusions (11).

The beginning of the concept of non-alcoholic fatty liver disease (NAFLD) was reported in the year 1980 by Ludwig et al. as a condition that can progress to cirrhosis without consumption of significant alcohol (12). The patients had diabetes mellitus and were obese. There have been significant changes in this nomenclature over the years giving the strong association of NAFLD with various metabolic factors. A new term for the condition “Metabolic (dysfunction) associated fatty liver disease (MAFLD)” was coined by Eslam et al. in 2020, that correlated with hepatic steatosis that could be diagnosed with at least two metabolic risk abnormalities, obesity, blood biomarkers or in the presence of T2DM (13). An important factor in this new nomenclature was to avoid any reference to alcohol in the MAFLD acronym. This has been further supported by the Asian Pacific Association for the Study of the Liver (APASL), multiple national societies including the Malaysian Society of Gastroenterology and Hepatology and a wide range of global stakeholders (11, 14, 15).

Several years later, the term MASLD was proposed, and its diagnosis can be done based on the patient meeting one of five cardiovascular risk factors, unlike MAFLD, which underlines a requirement that the patients meet two of seven parameters of metabolic dysfunction (5). MetALD is a term coined for patients with MASLD along with consumption of alcohol (140–350 g/week and 210–420 g/week for females and males, respectively).

**Figure 1:**
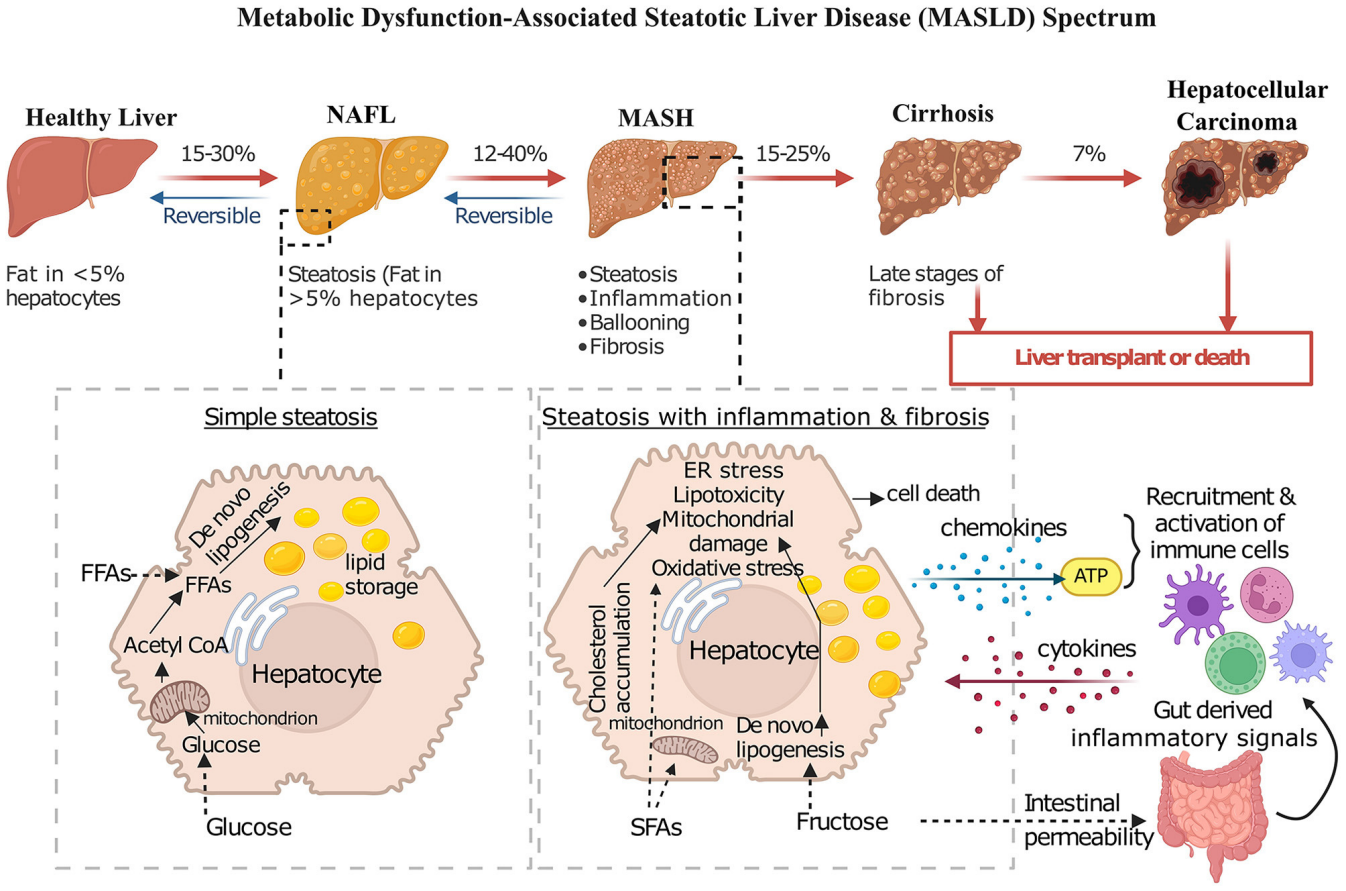
**Progressive stages of MASLD and its complications**. This diagram shows the progressive nature of MASLD, encompassing a spectrum of liver conditions ranging from simple steatosis to advanced stages such as MASH, cirrhosis, and hepatocellular carcinoma. In healthy individuals, fat content within hepatocytes typically remains low. In NAFL, fat accumulation increases, but the condition is often reversible with lifestyle modifications. MASH, however, involves not only fat accumulation but also inflammation, increasing degeneration of hepatocytes, and the formation of fibrosis. Cirrhosis, the most advanced stage, is characterized by extensive scarring that disrupts liver function, leading to serious complications like liver failure and increasing the risk of developing hepatocellular carcinoma. The diagram also depicts key pathological mechanisms contributing to MASH progression, including lipid accumulation due to increased uptake of free fatty acids and *de novo* lipogenesis (DNL), mitochondrial dysfunction, ER stress, oxidative stress, and inflammation mediated by immune cell activation and cytokine release.

The severe form of MASLD is MASH, a replacement of the term non-alcoholic steatohepatitis (NASH), characterized by the presence of lobular inflammation and ballooning of hepatocytes and is associated with a greater risk of fibrosis progression (Figure 1). A multi-society Delphi consensus statement on a new fatty liver disease nomenclature was published in 2023, thereby introducing the term metabolic dysfunction-associated steatotic liver disease (MASLD) and effectively letting go of the term NAFLD and MAFLD (16). The nomenclature was based on diagnostic criteria that was non-stigmatizing and aims to improve patient awareness.

Dysbiosis of the gut microbiota has been consistently linked to both obesity and T2DM—two metabolic disorders closely associated with MASLD. By examining the gut-liver axis as the central integrative pathway, this review explores how microbial dysbiosis mechanistically contributes to MASLD initiation and progression, and how hepatic metabolic dysfunction reciprocally alters gut microbiome composition. Our goal is to integrate essential factors such as obesity, insulin resistance, the gut-liver axis, immune system changes, and the role of the microbiome, providing a comprehensive overview of this increasingly common and complex metabolic disorder.

## Pathophysiology of MASLD/MASH

MASLD is characterized by excessive fat accumulation in the liver, with a spectrum ranging from simple steatosis to MASH and potentially cirrhosis (Figure 1).

The development of MASLD occurs in a coordinated fashion and was proposed earlier as a two-hit hypothesis (17). The first hit is the steatosis through *de novo* lipogenesis (DNL) in the liver which increases the insulin resistance (17, 18). The second hit means the progression from MASLD to MASH, representing a critical escalation in liver disease severity involving additional cellular and molecular stresses like endoplasmic reticulum (ER) stress, mitochondrial damage, oxidative stress [involving the production of reactive oxygen species (ROS)] (19, 20). Accumulation of saturated fatty acids (SFAs) increases DNL resulting from increased fructose uptake, or cholesterol accumulation in the ER leading to cellular stress (Figure 1) (17, 21). Increased fructose levels are also one of the key contributors to the progression of MASLD to MASH by increasing gut permeability, which initiates a cascade of inflammatory responses by releasing cytokines and promoting microbiota dysbiosis (22). This dysregulation is compounded by heightened activation of hepatic toll-like receptors (TLRs), changes in bile acid metabolism, and local alcohol production by gut microbes, all of which can exacerbate inflammation and tissue damage (23). Elevated blood ethanol levels in MASH patients further indicate the presence of alcohol-producing bacteria, which potentially elevate the production of ROS, adding another layer of hepatic inflammation and stress (24).

However, at present it is referred as the “multi-hit hypothesis” as the development and progression of the disease arises from a combination of factors that are interconnected and contribute to the advancement of the disease.

Lipogenesis is fuelled by the uptake of glucose and free fatty acids (FFAs) and their incorporation into lipid-synthesis pathways. In most cases, steatosis is an early event in MASLD, but it does not necessarily transition to MASH. Lipogenesis in the liver and lipolysis of the adipose tissue result in elevated circulating FFA and their metabolites which can induce inflammatory responses. This further activates TLR4 signaling that activates the NF-kB pathway; these are crucial to progression to MASH (5). This leads to inflammation, fibrosis and hepatocarcinogenesis (25). Beyond lipid accumulation, insulin resistance is important in MASLD as its presence leads to increased FFA in blood and lipid accumulation. Interleukins and cytokines are released by adipocytes and ROS are generated due to oxidation of excess FFA (26, 27). Oxidative stress in such conditions is a step forward during development of fibrosis in MASH. Another factor that contributes to progression of MASLD is autophagic dysfunction in cellular degradative organelles. Damaged mitochondria during MALSD, unable to undergo autophagy also contribute to build-up of ROS and lowering the defense system of the liver (28). Ultimately, dysbiosis of the gut microbiota plays a key role in driving hepatic fibrogenesis. Excess lipid accumulation compromises the gut barrier, and hence microbial toxins translocate into the bloodstream. The cumulative effect of these pathogenic processes promotes hepatic inflammation and triggers apoptosis of hepatocytes. These cellular damages lead to release of pro-inflammatory mediators such as ATP, extracellular vesicles, and chemokines, subsequently reinforcing the inflammatory process and leading to the development of fibrosis. Further, the prolonged inflammation may progress to cirrhosis and ultimately HCC.

## The interplay between MASLD, insulin resistance, and metabolic syndrome

**Figure 2:**
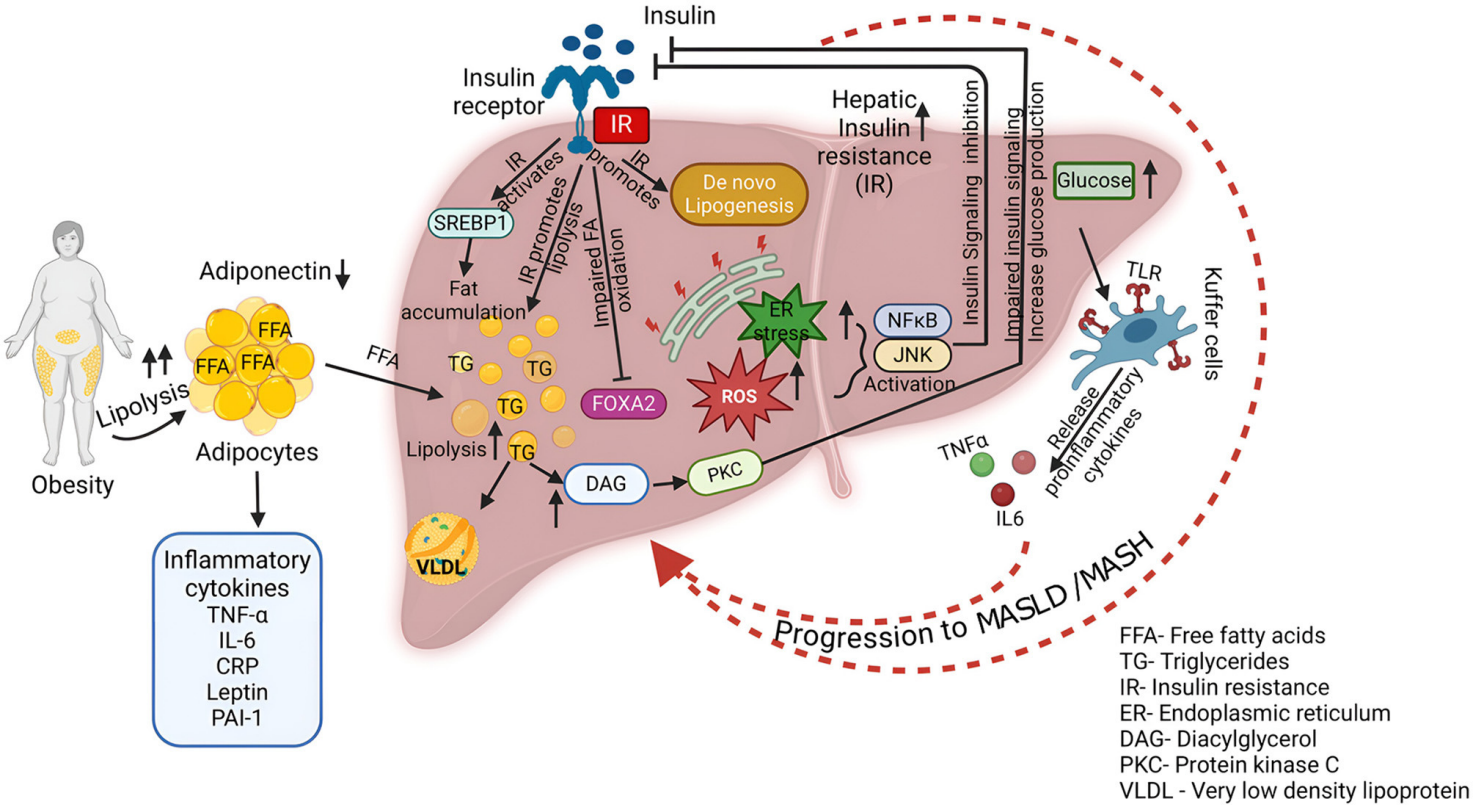
**Association of MASLD with metabolic syndrome**. This diagram shows the complex interplay of factors contributing to the development and progression of MASLD/MASH. Key processes include increased lipolysis in obese individuals, leading to elevated FFA levels. Insulin resistance impairs hepatic insulin signaling, promoting DNL, reducing fatty acid oxidation, and increasing glucose production. Excessive FFA influx, coupled with impaired fatty acid oxidation and increased DNL, results in triglyceride accumulation within the liver. This lipid accumulation triggers ER stress, leading to the activation of inflammatory pathways. Activated Kupffer cells release pro-inflammatory cytokines such as TNF-α and IL-6, exacerbating inflammation and tissue damage. Furthermore, increased fatty acid oxidation generates ROS, contributing to oxidative stress and hepatocellular damage.

MASLD is strongly associated with metabolic syndrome, which includes obesity, T2DM, dyslipidemia, and hypertension (Figure 2) (29–32). As the global prevalence of obesity and T2DM rises, so does the prevalence of MASLD, emphasizing its connection to metabolic dysfunction (33–35). Given its nature, the disease's progression is marked by insulin resistance (IR), particularly in the liver. Impaired ability of insulin to suppress endogenous glucose synthesis, 45–50% reductions in glucose disposal and a measure of whole-body insulin sensitivity are the indicative of hepatic insulin resistance (30, 36).

Obesity is widely recognized as a key factor in promoting systemic inflammation, which is closely linked to the development of IR (37). Specifically, abdominal visceral fat plays a significant role in both peripheral and hepatic IR in individuals with T2DM, while excessive subcutaneous fat in men has also been associated with IR in the liver and peripheral tissues (38). Adiponectin, an adipokine secreted by adipocytes, shows an inverse relationship with the amount of fat in the abdomen and liver, which is closely tied to both hepatic and peripheral IR (39, 40). Obesity activates various proinflammatory pathways, which includes elevated level of cytokines such as tumor necrosis factor-α (TNF-α), interleukin-6 (IL-6), C-reactive protein (CRP), plasminogen activator inhibitor-1 (PAI-1), and leptin (Figure 2) (37, 41). These molecules are the main causative factors in the pathophysiology of IR. Conditions of obesity or excessive nutrient intake induce ER stress, production of ROS, and accumulation of ceramides, which activate the NF-κB and JNK pathways, leading to insulin signaling inhibition (42–45). Inhibition of JNK1 and IKK-β (which activates NF-κB) in mouse models has shown improvements in IR both locally in the liver and systemically, highlighting the role of inflammation in exacerbating IR (46, 47). This mutual reinforcement of inflammation and IR creates a vicious cycle that worsens both conditions (48).

IR is considered a central mechanism in the development and progression of MASLD to MASH and cirrhosis, impairing the liver's ability to manage fat (49–51). The liver's supply of fatty acids, a primary source of triglyceride (TG) synthesis, comes from dietary fat, lipolysis in adipocytes, and DNL (Figure 2) (49). Both high-fat and high-carbohydrate diets promote fat accumulation in the liver, with FFAs from adipocytes playing a critical role (52). Normally, insulin suppresses lipolysis, but in the state of IR, this suppression is impaired, leading to excessive FFAs that accumulate in the liver (53, 54). Additionally, hyperinsulinemia, a hallmark of IR, further exacerbates liver fat accumulation by promoting DNL through the activation of sterol regulatory element-binding protein 1c (SREBP-1c) (55). In addition to increasing liver fat synthesis, hyperinsulinemia elevates triglyceride production and promotes very-low-density lipoprotein (VLDL) synthesis. However, insulin resistance impairs fatty acid oxidation, a process regulated by forkhead box protein A2 (FOXA2) (55). In normal conditions, FOXA2 promotes lipid metabolism, but it remains inactive in hyperinsulinemic states, leading to the accumulation of fat in the liver. As part of the liver's adaptation to excessive FFAs, mitochondrial respiration rates increase, yet excessive fatty acid oxidation results in oxidative stress and hepatocellular damage, contributing to the progression to MASH (56). While IR is a major cause of fatty liver, some evidence suggests that fatty liver itself can exacerbate IR (57, 58). The influx of FFAs into the liver leads to an abnormal increase in long-chain fatty acyl-CoA and diacylglycerol (DAG), which in turn activates protein kinase C-δ (PKC-δ) (59). This activation disrupts insulin signaling and promotes glucose production in the liver. FFAs also activate the IKK-β and JNK pathways, further exacerbating IR via PKC-θ activation (60). These inflammatory pathways, activated by fatty acid influx, play a central role in the vicious cycle of liver fat accumulation and IR. In MASLD, a paradoxical relationship exists in which increased DNL coexists with inappropriately elevated gluconeogenesis despite hyperinsulinemia. This has led to the concept of pathway-specific hepatic insulin resistance, where the insulin activation pathway involving protein kinase B/forkhead box protein O1 is inhibited, while the SREBP-1c pathway remains activated (61). Activation of carbohydrate response element-binding protein (ChREBP) also induces an increase in precursors of DNL and an increase in enzymes that further aggravate hepatic steatosis, especially under exposure to lipogenic substrates (62).

As MASLD progresses, hepatic inflammation, particularly involving M1 macrophages (Kupffer cells), becomes a key factor in disease progression (63). These macrophages, when activated by TLR ligands and interferon-γ, release proinflammatory cytokines such as TNF-α and IL-6, which contribute to the progression of MASLD and systemic IR by modulating other immune cells (64) (Table 1).

**Table 1:** Engagement of different cells of the immune system in severity and progression of MASH.^*^

**Name of immune cell**	**Engagement and role of cell in MASH**	**Reference**
B cells	Pro-inflammatory; they accumulate in livers of patients with MASH and high lobular inflammation & cirrhosis; B2 cell depletion takes place	(20, 305)
Dendritic cells	Capable of participation in local inflammation via engaging Toll-like receptors; cDC1 are known to increase in MASH patients.	(306–308)
Inflammatory cytokine IFNγ (T_H_1 associated cytokine)	Decreased levels inhibit liver fibrosis; maybe caused due to decrease in infiltration of macrophages/Kupffer cells and suppressing the inflammatory response.	(309)
TNF-α and TNF receptor 1 transcripts	Levels increased in patients with steatohepatitis.	(310)
CD8^+^T cells	Produce IFNγ, TNF and perforins; numbers of CD8^+^T cells are increased during MASH; these promote hepatocellular carcinoma through interactions with hepatocytes. Natural killer cells along with CD8^+^T cells.	(311)
LIGHT	TNF cytokine family member, expressed on lymphocyte acting as a key regulator of enzyme that control lipid metabolism. LIGHT signaling is shown to positively regulate hepatic lipid uptake.	(312)
iNKT cells	Levels increased in patients with MASH; promote liver fibrosis by promoting expression of osteopontin (OPN; pro-inflammatory cytokine & extracellular matrix protein). OPN promotes Hedgehog pathway activity and progression of fibrosis.	(313, 314)
Platelets	Activation and adhesion of platelets promote MASH, liver steatosis and promote accumulation of inflammatory cells in a glycoprotein dependant manner.	(315)
Neutrophils	Hepatic infiltration of neutrophils is seen during MASH and production of ROS, cytokines, proteases and NETs (neutrophil extracellular traps).	(316)
Macrophages	Kupffer cells, the tissue dominant macrophages are lost during MASH progression due to lipotoxic stress and they lose their ability for self-renewal.	(317)

^*^Understanding the role of various immune system cells in MASH offers valuable potential for identifying new therapeutic targets and advancing more effective treatment strategies for this complex and increasingly common liver disease (318–320). However, preclinical evidence is still needed before considering the use of these cell populations as therapeutic targets in MASLD & MASH.

## Gut microbiome and MASLD: a key connection

Building on the understanding of MASLD and its progression to MASH, the role of the gut microbiome has emerged as a significant factor in disease pathogenesis. Reviewing microbial signatures with respect to various stages of progression of liver disease would be very useful for predicting biomarkers as well for designing therapeutic approaches (Table 2). Gut microbiota, the collective genome of gut-residing microbes, is increasingly recognized as an environmental factor that influences metabolic health by impacting energy balance, inflammation, and IR (65–70). It has been demonstrated in both mice and human studies that obesity and T2DM are associated with changes in gut microbiota, though it is not clear whether these are the cause or effect of the underlying metabolic changes (71). Specifically, obese people and mice have fewer *Bacteroidetes* and more *Firmicutes* compared with their lean counterparts. More importantly, the relative proportion of these two major bacterial divisions is positively correlated with body weight, *i.e*., a higher proportion of *Firmicutes* and a lower proportion of *Bacteroidetes*. This altered ratio is associated with increased energy harvest from food, potentially exacerbating obesity (71). High-fat diets (HFD) in mice have also been shown to reduce beneficial bacteria such as *Bifidobacteria*, which improve mucosal barrier function, reducing gut permeability and inflammation (72). Such microbiota shifts promote inflammation and metabolic dysfunction, creating a cycle that worsens insulin resistance and metabolic syndrome (71, 73).

**Table 2:** Microbial species distribution across different stages of MASLD and MASH.

**Disease stage**	**Microbial species changes**	**References**
**Initial MASLD development**		
Stool microbiome profile	• ↑ *Lachnospiraceaebacterium*609^1^	(91)
	• ↑ *Barnesiellaintestinihominis*^1^	
	• ↑ *Lactobacillus* spp. (*L. gasseri, L. taiwanensis*)^1^	(94)
**Disease progression**		
Mild/moderate MASLD	• ↑ *Eubacteriumrectale*^2^	(114)
	• ↑ *Bacteroidesvulgatus*^2^	
	• ↑ *Firmicutes* (dominant phylum)^2^	
	• ↓ *Rikenellaceae*^2^	(4, 136)
	• ↓ *Ruminococcaceae*^2^	(4, 136)
	• ↑ *Enterobacteriace*^2^	(4)
	• ↑ *Dorea* ^2^	(4, 136)
	• ↑ *Peptoniphilus* ^2^	(4)
	• ↓ *Anaerosporobacter* ^2^	(4)
	• ↓ *Coprococcus*^2^	(4)
	• ↓ *Faecalibacterium* ^2^	(4)
Advanced fibrosis	• ↑ *B*.*vulgatus*^3^	(114, 321)
	• ↑ *Escherichiacoli*^3^	
	• ↑ *Proteobacteria* phylum^3^	
	• ↑ *Gammaproteobacteria*^3^	
	• ↑ *Prevotella* spp.^3^	
	• ↓ *Firmicutes*^3^	
	• ↓ *Ruminococcus obeum* CAG:39^3^	
	• ↓ *R*.*obeum*^3^	
	• ↓ *E*.*rectale*^3^	
MASH	• ↑ *Bacteroides* spp.4	(75)
	• ↓ *Prevotella* spp.4	

^1^Species overrepresented in stool with potential to induce MASLD. ^2^Characteristic of early disease stage. ^3^Associated with disease progression ^4^Markers of inflammatory progression.

**Key Finding:** Advanced MASH fibrosis is characterized by a significant decrease in Gram-positive Firmicutes and increase in Gram-negative Proteobacteria (including E. coli). The presence of lipopolysaccharides and endotoxins from gram-negative bacteria contributes to hepatic fibrosis progression (322, 323) (Symbols: ↑, increased abundance; ↓, decreased abundance).

The genome of the microbiome bears information to coding of several enzymes that are absent in the human host, and they cooperate with contribution to individualized traits in the host (74). Obesity and improper eating habits have been significant co-occurrences with MASLD; however, presence/absence of many bacterial species have altered the microbiome in such conditions. Studies have shown increased abundance of bacteria from the *Proteobacteria* phylum, especially within the *Enterobacteriaceae* family and the *Escherichia* genus in individuals with MASLD and MASH compared to their healthy counterparts (24). An increased abundance of *Bacteroides* genus was reported in MASH (75). Pathogenesis of MASH has been influenced by increased fecal content of deoxycholic acid, raffinose, choline, D-pinitol and stachyose in patients.

A 16S rRNA cohort study of biopsy proven Asian population was done to explore microbial markers for assessment of severity of fibrosis between obese and non-obese subjects (76). An increase in levels of total bile acid, especially primary bile acids and ursodeoxycholic acid (UDCA), and propionate levels was seen in stool samples of subjects with worsening fibrosis. A dominance of bacterial population of *Veillonellaceae* was found in non-obese individuals with MASLD. Six genera of Gram- negative bacteria (*Megasphaera, Veillonella, Dialister, Allisonella, Anaeroglobus*, and *Negativicoccus*) are a part of the family *Veillonellaceae* and these are known to produce propionate-and can utilize lactate as a substrate. It has been proposed that accumulation of these populations lead to more propionate production that is absorbed into the liver and hence progression to MASLD. On the contrary, *Ruminococcaceae* members were found to decrease in numbers in fibrosis in MASLD non-obese patients and are responsible for maintaining homeostasis of the gut microbial environment (Table 2). *R. faecis* exerted a protective effect on liver damage. It was concluded that assessment of gut microbes and stool metabolites could be used for diagnosis of fibrosis in non-obese subjects with MASLD.

The impact of diet on gut microbiota composition further influences MASLD progression, as evidenced by studies showing that *Bacteroides* thrive on high-fat animal-based diets, while *Prevotella* is more prevalent with plant-based polysaccharide diets (77, 78). Contents of branched chain fatty acids due to *Bacteroides* population produced by fermentation of amino acids are correlated with insulin resistance that further gives impetus to development of MASH. A study conducted with stool samples of mice with MASLD-MCC revealed that oral administration of a species of *Bifidobacterium pseudolongum* was successful in preventing hepatocellular carcinogenesis (10). Ramos et al. performed a detailed analysis of the gut microbiome of patients with biopsy proven MASLD and their study concluded an enrichment of *Parabacteroides distasonis* and *Alistipes putredenis* species in MASLD patients. They also found that *Prevotella copri*, was a dominant species for MASLD disease progression also linked to higher intestinal permeability (79). Iljazovic et al. (80) demonstrated that *Prevotella* populations, previously associated with colitis in animal studies, can worsen intestinal inflammation and potentially lead to systemic autoimmune responses condition (24). This may occur by reducing IL-18 production, which further intensifies gut inflammation. Increased population of *Prevotella* has been linked to mucosal sites with inflammation and increase in T-helper type 17 cells mediating this process (81). These bacteria have also been implicated in activation of TLR-2, that lead to production of Th17- polarizing cytokines by antigen presenting cells such as IL-23 and IL-1. They mediate spreading of inflammatory mediators and bacterial products. Additionally, elevated blood ethanol levels in MASH patients indicate the presence of alcohol-producing bacteria, which contributes to the production of ROS, further increasing hepatic inflammation and oxidative stress.

In patients with MASH, gut microbiota changes extend beyond metabolic influences to play a direct role in liver pathology. The composition of gut microbiota can affect the liver due to the portal circulation of venous blood from the gut to the liver. Changes in gut microbes and their derived products can have effects on systemic and hepatic immunity, inflammation, and liver architecture and function (82–88). It is now recognized that MASLD is the hepatic expression of metabolic syndrome and given the association of gut microbiota with obesity and insulin resistance, several studies have sought to investigate the role of gut microbiota in MASLD (89–91). The most abundant species traced during advanced fibrosis are *E.coli* and *Bacteroides vulgatus* (4, 79). *B. vulgatus* is known to increase with obesity, increment in BMI, Hb1Ac and insulin resistance (92). The bacterial populations in advanced stages of cirrhosis undergo a major transformation, with an increase in pathogenic bacteria and decrease in beneficial bacteria. A reduction in *Faecalibacterium prausnitzii*, an anti-inflammatory species has been reported in subjects with cirrhosis (92, 93). Its decrease is also seen in other conditions such as obesity, T2DM and bowel diseases (4).

Several animal studies have analyzed the changes in gut microbiota in response to high fat feeding (73, 91, 94). Mice deficient in the hormone leptin are called ob/ob and the ones deficient in leptin receptor are called db/db that mimic conditions of obese and diabetic strains respectively. In the ob/ob mouse, the changes in microbiota occurred rapidly following the administration of a high fat diet resulting in an increase in proportional weight in *Firmicutes* and a decrease in *Bacteroidetes* (71, 85) and an increase in intestinal permeability. A decrease in *Bacteroidetes* was also seen in the TLR4 knockout mouse whilst there were no differences in wild type mice. More recent studies have found that high fat diets in mice lead to increased presence of gram-negative bacteria, malonaldehyde (MDA) modified end products, and reduced defensin expression. An MDA rich environment may cause induction of bacterial cytolysins and affect antimicrobial defense mechanisms in the gut. Several studies have examined diet-induced changes in gut microbiota in mice strains fed a high fat or high sucrose diet and, in each case, a significant proportional increase in *Firmicutes* and decrease in *Bacteroidetes* was seen (95–99). High fat or high sucrose feeding also led to increased *Dorea* and *Eubacterium rectale* and decreased *Bifidobacterium* (100–103). To date, the only human dietary intervention study found that weight loss by energy restriction with a Mediterranean or low-fat diet increased *Firmicutes* and reduced *Bacteroidetes*: *Eubacterium rectale* in obese individuals (101, 104–106). This is important as it shows that specific changes in human gut microbiota can be linked to dietary patterns and may be relevant to the etiology of MASLD (90).

## Gut barrier dysfunction and endotoxemia

Intestine is one of the most crucial internal barriers and its disturbance leads to an immune response as bacterial products pass through the gut, and evidence shows that the immune systems of patients with MASLD are primed toward a pro-inflammatory state. This explains why the severity of MASLD is often associated with the presence of an inflammatory state, and the immune response is closely linked with the mechanism of liver damage and inflammation. Intestinal permeability refers to ability of the extracellular barrier to allow any exchange between tissues and intestinal lumen. The gut barrier limits passage of potentially pathogenic molecules and microorganisms to the systemic circulation (107). Passage of bacteria and their products from the gut lumen to the bloodstream and liver and spleen is known as intestinal bacterial translocation. Livers of Healthy individuals have an exposure of small bacterial products such as lipopolysaccharides (LPS), a dominant molecule on surface of Gram-negative bacteria. Increased levels of LPS are found in patients of inflammatory diseases. Hepatic inflammation is resultant of a complex interaction of Kupffer cells, neutrophils, hepatocytes and sinusoidal cells. Metabolic dysfunction in the liver takes place because of interaction of hepatocytes and Kupffer cells with pathogen-associated molecular patterns (PAMPs) and damage-associated molecular patterns (DAMPs) and initiating a series of inflammatory events.

**Figure 3:**
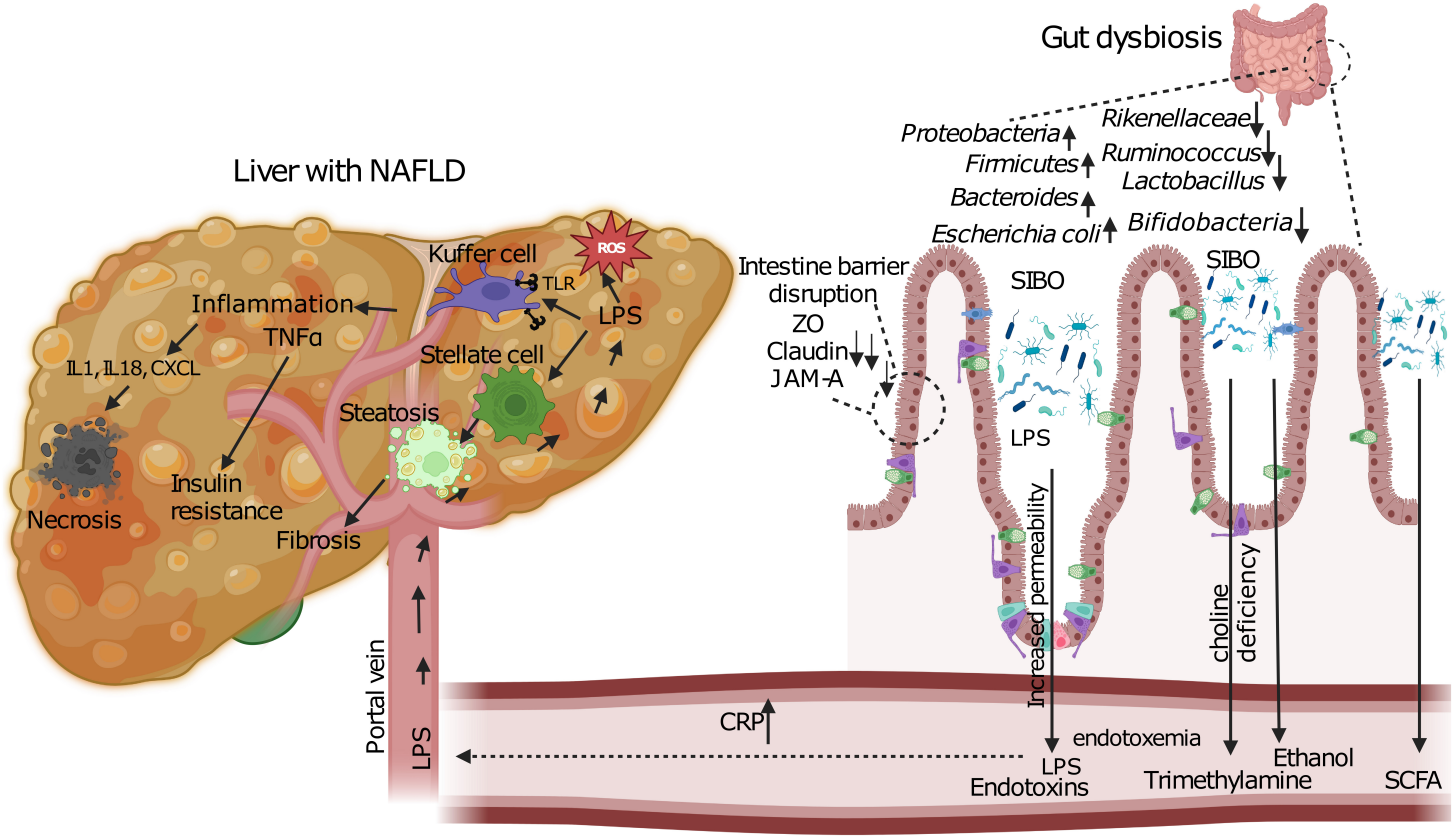
**Pathogenic mechanisms linking gut dysbiosis to MASLD progression**. Intestinal dysbiosis and SIBO lead to barrier dysfunction through disruption of tight junction proteins (ZO, Claudin, JAM-A). This results in increased intestinal permeability and translocation of bacterial endotoxins (LPS) via the portal vein. In the liver, LPS activates Kupffer and stellate cells, triggering inflammatory cascades (TNFα, IL1, IL18, CXCL) and oxidative stress (ROS). Concurrent metabolic alterations include increased ethanol production, altered SCFA metabolism, and choline deficiency due to enhanced bacterial conversion to trimethylamine. These pathways collectively promote hepatic steatosis, inflammation, and fibrosis characteristic of MASLD.

Increased blood endotoxin levels have been detected in patients with MASLD on comparison with healthy liver controls which also suggests that these could be used an indicative biomarker for progression of liver disease (108). The LPS present on the outer cell membrane of Gram-negative bacteria in the intestine constitute these endotoxins that can induce inflammatory activities. Dysbiosis coupled with disturbance of the intestinal barrier leads to release of endotoxins from the lumen of the gut into the circulation and it enters the liver via the portal vein (Figure 3). Increase in levels of endotoxins is also coupled with a simultaneous increase in C- reactive protein (CRP) that is considered as a marker for systemic inflammation (Figure 3). Excessive growth of aerobic and anaerobic gram-negative bacteria is known to cause a condition called small intestinal bacterial overgrowth (SIBO) (109). This contribution of SIBO can be due to increased oxidative stress, insulin resistance, increased ethanol production and by modulating choline metabolism by promoting the excessive conversion of choline into trimethylamine (TMA) by gut bacteria, leading to potential choline deficiency and contributing to the development of MASLD. Increase in gut permeability due to disruption of the tight cell junctions in SIBO affected individuals would lead to entry of bacteria and their products—endotoxins. This in turn induces expression of nuclear kappa B expression that activates TLR-4 and proinflammatory cytokines such as TNF-α and IL-6 and IL-8 (Figure 3) (110, 111).

## Impact of dysbiosis in MASLD

Gut dysbiosis disrupts the bile acid metabolism pathway, which in turn causes dysfunction of the gut-liver axis (112). Evidence showing an increase in potential harmful bacteria (e.g. *Escherichia coli*, and *Bacteroides*) and a decrease in beneficial bacteria (e.g. *Bifidobacteria* and *Lactobacillus*), strengthened the fact that dysbiosis is associated with MASLD (113–116). Studies have shown MASLD alterations like hepatic triglyceride elevated levels, upregulation of genes related to lipid uptake and lipogenesis in germ free mice upon fecal matter transplant (FMT) from hepatic steatosis suffering obese mice (91). In another study, it was found that inflammasome-mediated gut dysbiosis can cause hepatic steatosis in wild-type mice when cohoused with MASH-affected mice (117). The metabolites produced by gut microbiome are essential component that can modulate the pathophysiology of MASLD and MASH. One of the most common metabolites produced by gut bacteria in response to dietary fiber breakdown is SCFA which plays a crucial role in maintaining metabolic, nervous, and immune system (118). By influencing host epigenetics, activating G protein-coupled receptors, and preventing pathogenic microbial infections, SCFAs function as vital mediators between the gut microbiota and the host, acting as energy substrates for intestinal epithelial cells and preserving homeostasis in host immune and energy metabolism (119). Acetic acid, propionic acid, and butyric acid are the most common SCFA accounting for 90–95% of the colon's total SCFA content (118). The acetate boosts liver fat oxidation by facilitating changes in mitochondria and activating AMP-activated protein kinase (120), while propionate may promote the release of leptin, which helps to suppress the formation of new lipids (121). Butyrate is primarily utilized by colon cells as their main energy source and displays anti-inflammatory properties (122). Butyric acid has also the ability to hinder the activation of ChREBP and SREBP-1, then, suppress the process of lipogenesis (123). Researchers have found in high-fat-fed mice, commensal microbe that produces acetate can suppress MASLD progression by modulating free fatty acid receptor 2 (FFAR2) signaling in the liver (124). Several other studies have demonstrated that butyrate can regulate gut microbiota, hepatic Glucagon-like peptide-1 (GLP-1) receptor expression, TLR4 pathways and intestinal tight junctions thus attenuating the development of MASLD (125–128). Decreased production of butyrate results in increased intestinal inflammation, increased gut permeability, endotoxemia and systemic inflammation (129). In addition to SCFA, bile acid and ethanol are other metabolites that plays a vital role in MASLD. Preclinical trials have shown that microbiota derived endogenous ethanol can accelerate liver steatosis and inflammation (24, 130). Additionally, there are evidence showing increased level of blood ethanol in MASLD patients (131). Gut microbiota is also involved in Bile acid metabolism. They have the capacity to convert primary bile acid into secondary bile acid. However, due to decreased abundance of related bacteria this ability is compromised in case of MASLD (132). By targeting genes linked to fatty acid synthesis and oxidative stress, a lower amount of deconjugated bile acid can further reduce taurine production and cause hepatic steatosis and inflammation (133). In addition, the receptor for bile acids, farnesoid X receptor (FXR) is found to be downregulated in MASLD (132). The decrease in the level of intestinal FXR is correlated with decrease in the secretion of an enterokine that regulate synthesis of hepatic bile acid fibroblast growth factor 15/19 (FGF15/19), which in turn can reduce liver steatosis (134). Amino acids and choline are some other gut microbial metabolites that are reported to modulate MASLD (129).

Diet plays a pivotal role in shaping the composition and function of the gut microbiome. A Western-style diet, rich in fats and refined carbohydrates, has been shown to induce dysbiosis and compromise gut barrier integrity. Interestingly, it has been found by investigators that obese infant mice with a western diet have excess weight gain and accelerate the progression of MASLD (135). It is hypothesized that onset of MASLD is triggered by high fat diet which induces an increase in FFAs and LPS which are derived from the gut anaerobic bacteria (114, 136). Endotoxemia occurs when there are elevated levels of LPS in the blood and is commonly observed in states of obesity, insulin resistance and in MASLD patients. Increased endotoxemia occurs due to higher levels of gut derived LPS and translocation of bacteria, particularly due to a high fat diet. In response to LPS, Kupffer cells, which are resident liver macrophages, and hepatic stellate cells are activated and release pro-inflammatory cytokines, this further elevates insulin resistance and promotes hepatic inflammation and fibrosis, thus having a central role in MASLD progression (137–139). LPS also induces fat accumulation in the liver and ROS production. Kupffer cells along with liver sinusoidal endothelial cells (LSECs), hepatic stellate cells (HSCs), and local immune cells, specifically unconventional T cells, natural killer (NK) cells, and hepatic dendritic cells make up the nonparenchymal liver cells, which are significant chemokine sources and responders. PAMPs or DAMPs are released when hepatocytes are damaged, whether by infection or other causes. These signals attach to TLR4 and other TLRs on Kupffer cells and trigger the release of proinflammatory cytokines, such as TNF, chemokines, and reactive oxygen and nitrogen species (140). TNF released by Kupffer cells is thought to play a key role in exacerbating liver damage, primarily by causing hepatocyte death but also by degrading the hepatic microcirculation by causing endothelial cells to swell and become activated, which leads to sinusoidal platelet aggregation and makes it easier for peripheral immune cells to enter. Interleukin (IL)-1β and CXC chemokines, including CXCL1, CXCL2, and CXCL8 (IL-8), are secreted by activated Kupffer cells. Key chemokines CXCL1, CXCL2, and CXCL8 draw neutrophils primarily through the chemokine receptors CXCR1 and CXCR2, which release proteases and ROS, causing hepatocyte necrosis (141).

## Therapeutic approaches

The existing diagnosis of MASLD relies on clinical evaluations as well as biopsy results, with liver biopsy being the only diagnostic method that can accurately determine its severity (142). Nonetheless, this invasive technique carries the risk of serious and potentially fatal complications. As a result, effectively forecasting and promptly taking measures to avert the onset of MASLD continues to be a challenge. Several studies which focus on blood biochemical markers, gut microbiota, and fecal SCFAs, uncover a close association between gut microbiota and the progression of MASLD, thereby improving its clinical diagnosis (142–144). While we emphasizes on improving clinical diagnosis using microbial signatures, we acknowledge that future validation studies should also include head-to-head comparisons with established non-invasive tests such as FIB-4 and elastography to determine the relative diagnostic performance and clinical utility of microbiome-based approaches.

As discussed, gut–liver axis is an important bridge between gut and liver. The dysbiosis/malfunction of the gut–liver axis plays one of the most important roles in the onset and progression of MASLD by altering the intestinal permeability, increasing the level of portal toxic metabolites, promoting hepatic inflammation. Thus, microbiota based pharmacological modulation of gut–liver axis is an emerging and promising therapeutic method for MASLD treatment (145–148). There are numerous pathways by which microbiota can affect liver health, and several approaches that have been proposed to target these in order to improve liver health (148–153). There are several drug candidates that are in later stage clinical trials which includes PPAR agonists, anti-fibrotic therapies, anti-inflammatory agents, antioxidants, and treatments targeting the gut-liver axis (154). The gut microbiota can be therapeutically modulated through several approaches, including antibiotic therapy, probiotic supplementation, prebiotic administration, synbiotic interventions (155, 156) and FMT (157, 158). Obesity and T2DM are linked to substantial compositional and functional alterations of the gut microbiota. Therefore, modulation of the gut microbiota represents an attractive approach for the management of diabetes in the context of MASLD (68, 91, 159, 160).

### Modulation of gut microbiota

Gut microbiota may be altered using prebiotics, probiotics or their combination known as synbiotic. According to FAO/WHO, Probiotics are live non-pathogenic microorganisms, which when administered in adequate amounts, confer a health benefit on the host (161, 162). The most widely used bacterial populations are of *Lactobacillus, Bifidobacterium* and *Streptococcus* that are capable of suppressing growth of Gram-negative pathogens (163, 164). These beneficial bacteria can reduce lipid deposition, endotoxemia, oxidative stress, and inflammation by regulating the expression levels of TNF-α, NF-κB, and collagen (162). Improving the gut barrier is the primary way that probiotics protect against MASLD in the gut–liver axis. *Lactobacillus rhamnosus, L. acidophilus, L. plantarum*, and *Streptococcus thermophilus* are a few probiotics that have shown the ability to activate tight junction proteins to improve the intestinal permeability (165). A recent randomized controlled trial has shown that probiotics stabilizes the mucosal immune function that in turn protects the MASLD patients from increased intestinal permeability (166). A variety of probiotics, particularly well-known like *Lactobacillus, Bifidobacterium*, and *Streptococci*, have been studied clinically in relation to the prevention and treatment of MASLD. Wong et al. treated MASH patients for 6 months with a variety of probiotics and discovered that the subjects receiving probiotics had a considerably lower liver fat level than the placebo group (167). According to clinical data, probiotics can help MASLD patients' liver histology and liver injury indices like alanine aminotransferase (ALT) and aspartate aminotransferase (AST) (168). But according to a different clinical study conducted during the same time, giving multiple-strain probiotics to MASLD patients only improved liver steatosis and not liver enzymes (169). Probiotics have consistently been shown to reduce liver enzyme levels (e.g., ALT, AST) in MASLD patients, but improvements in liver histology like inflammation, fibrosis, or steatosis have not been demonstrated in biopsy-confirmed settings. Several meta-analyses confirm this pattern (162, 169–177). A 2024 network meta-analysis of 37 randomized controlled trials (RCTs, n=1,921) found that probiotics significantly lowered ALT and AST, and improved liver stiffness and steatosis based on elastography and controlled attenuation parameter (CAP) scores but did not show histological resolution (178). Similarly, a 2019 meta-analysis reported significant reductions in liver enzymes and steatosis by ultrasound, but histological effects were unassessed (162, 174, 179). Most trials rely on biochemical or imaging measures (e.g., ultrasound or FibroScan), and rarely include sequential liver biopsies to confirm tissue-level changes. Probiotics effectively lower liver enzymes in MASLD without proven histological improvements, likely due to non-tailored approaches. Personalized microbiome modulation using patient-specific microbial profiling, strain selection, prebiotic support, and mechanistic biomarkers offers a promising path to bridge this efficacy gap. Ongoing research in precision microbiome interventions (pharmacomicrobiomics, host-microbe profiling) will be critical for developing such customized therapeutic strategies (180).

Prebiotics are non-digestible food ingredients that have beneficial effects on the host by selectively stimulating the growth and/or activity of one or a limited number of bacteria in the colon, and thus improving host health (181, 182). Prebiotics are capable of increasing activity of good bacteria and resisting growth of detrimental species. Fructooligosaccharides (FOS), inulin, transgalactooligosaccharides (TOS), and lactulose are examples of common prebiotics. Prebiotics are a safe and efficient way to control the gut microbiota since they can boost the growth and activity of probiotics (183). Prebiotics can prevent the growth of harmful bacteria like *Salmonella enteritidis, Klebsiella pneumoniae*, and *Escherichia coli* while simultaneously activating the advantageous bacteria (184). This characteristic can enhance the gut barrier, support gut microbial homeostasis, and ultimately slow the advancement of MASLD. Through fermentation, prebiotics can also protect against MASLD by producing SCFAs, which have been shown to protect against MASLD and the gut-liver axis (183). Larch wood arabinogalactan (LA-AG), a novel complex soluble dietary fiber was discovered by Sun et al., as a potential prebiotic. By promoting the fermentation of organic acids, LA-AG was able to reduce the activity of harmful bacteria and enhance intestinal health (185). Therefore, by controlling the gut-liver axis, LA-AG may be effective in preventing MASLD.

In a clinical trial Bomhof et al. (186) showed, using oligofructose as an example, that giving patients with MASH a supplement of the prebiotic can improve their liver steatosis and non-alcoholic fatty liver activity score (NAS) (186). Furthermore, according to a meta-analysis prebiotic treatment can enhance anthropometric and biochemical parameters such as body mass index (BMI), ALT, AST, fasting insulin, and insulin resistance in individuals with MASLD (187).

Apart from probiotic and prebiotics, their combination called synbiotic can be used that is capable of boosting metabolism of heathy bacteria and modulate the gut microbiome (161). Several studies have shown the protective effect of synbiotics on liver and cardio related disorders (171, 179, 188–190). More studies to define the health benefits of pre and probiotics in the context of MASLD and T2DM are required. If these therapies are shown to be beneficial, an important issue will be the best strains of probiotics or types of prebiotics to use, and the optimal duration of therapy. This microbe directed therapy for MASLD, and diabetes could also involve the use of antibiotics with selective action in the intestine, although this is unlikely to be an attractive strategy for patients or doctors. The idea that antibiotics can reduce the effects of microbiota and their metabolites on host metabolism via the gut–liver axis is the foundation for their use in the treatment of MASLD. Preclinical trials have shown that by inhibiting gut bacteria, antibiotics can control the amount of portal secondary bile acid, reducing liver fibrosis and inflammation and preventing the progression of MASLD (191). Another study demonstrated that neomycin and polymyxin B can significantly lower hepatic lipid build-up by decreasing the translocation of endotoxin in a MASLD mouse model (192). In a Phase II clinical trial, the powerful next-generation macrolide antibiotic Solithromycin was shown to lower the ALT and NAS of MASH patients (193). There are several studies in animal models demonstrating that broad-spectrum antibiotics can prevent and reverse MASLD, although the side effects of long-term antibiotic use are considerable (194–196). Antibiotics should be used with caution as they may eradicate certain bacterial species linked to good health and result in the emergence of some antibiotic-resistant bacteria (197).

An exciting potential future alternative is the use of FMT from a healthy lean donor to a patient with MASLD and obesity or diabetes (174, 198, 199). FMT is an effective therapeutic option for liver and metabolic diseases associated with intestinal microbiota dysbiosis (85, 198, 200). There have been several studies demonstrating the therapeutic effects of FMT on ulcerative colitis, T2DM and patients, which were associated with improved insulin resistance, restored healthy microbiota, and normalized blood lipid levels (201–205). Several investigations have demonstrated that FMT is an effective bacteriotherapy for MASLD as well. Zhou et al. discovered in an early preclinical investigation that FMT might reduce High fat diet (HFD)-induced MASH by enhancing the gut barrier, raising SCFA levels, and controlling gut microbiota (206). Another study in 2021 by Zhang et al., demonstrated that germ-free (GF) mice receiving FMT had less hepatic lipid accumulation and inflammation than normal chow-fed animals in contrast to mice fed high-fat/high-cholesterol (HFHC) and receiving FMT (207). Recent human trials have also shown that FMT can lower intestinal permeability and hepatic steatosis in MASLD patients, which is in line with animal investigations (208). However, some side effects, like bacteremia and perforations, have still been documented in FMT (209). Therefore, additional clinical trials must be carried out to increase the effectiveness and lower the negative effects of FMT treatment in MASLD/MASH.

Microbiota-based therapies, such as probiotics and FMT, are being explored as adjunct treatments for MASLD, primarily due to the gut-liver axis's role in disease progression. However, current evidence reveals several critical limitations.

For probiotics, therapeutic effects vary widely depending on the strain, dose, and treatment duration. While some studies report reductions in liver enzymes and steatosis, results are inconsistent, and few trials assess histological endpoints (174, 178, 210). Moreover, the adult gut microbiome exhibits strong ecological resilience, often reverting to its original state after probiotic intervention (174), limiting long-term efficacy. There's also no consensus on optimal strains or treatment regimens (211).

FMT shows some promise in early MASLD trials (212, 213), but results are mixed. Engraftment of donor microbes is unpredictable and often influenced by host factors, such as baseline microbiota composition and diet (214). Safety concerns have also been raised—cases of extended spectrum beta lactamase (ESBL)-producing *E. coli* infection following FMT prompted FDA safety alerts (215), underscoring the need for rigorous donor screening.

Furthermore, both therapies lack standardized protocols regarding delivery method, donor/strain selection, and outcome measurement. Most trials are short-term and fail to evaluate long-term outcomes like fibrosis reversal. Given that MASLD is a complex, multifactorial disease, targeting the microbiome alone may be insufficient without concurrent lifestyle or metabolic interventions (4, 216).

In conclusion, while microbiota-based therapies hold promise as adjunctive treatments for MASLD, they are currently limited by inconsistent efficacy, methodological heterogeneity, safety concerns, and incomplete understanding of long-term outcomes. Well-designed RCT's with standardized protocols, mechanistic endpoints, and extended follow-up periods are urgently needed to clarify their role. Until such data are available, these therapies should be considered experimental and used with caution in the clinical setting.

### Other promising agents as adjunctive therapy

#### Using FXR agonists

FXR agonists are a class of drugs that have been reported to decrease hepatic steatosis and improving insulin sensitivity and hence a promise in treatment of various gastrointestinal diseases (217). FXRs are nuclear receptors present in liver, kidney, intestine, pancreas and adipose tissue and are actively involved in bile acid, lipid and glucose metabolism and inflammation. The FXR agonists bind to the receptors and activate them and regulate target genes involved in the biological pathways. These agonists have been a part of successful clinical trials which showed that they assist in improvement in liver inflammation as well as insulin sensitivity. These receptors are activated endogenously by bile acids and are regulators of bile acid production, conjugation, and transport.

Several FXR agonists that have been assessed in clinical trials and their effects are:

- Obeticholic Acid (OCA): A derivative/synthetic variant of bile acid, it improves insulin sensitivity, liver inflammation, hepatocellular ballooning and reduces fibrosis (218). When bound to the FXR receptors, lipophilic bile acids decrease gluconeogenesis and triglycerides in the liver, promote insulin sensitivity. This also increases the expression of hepatic scavenger receptors (SRB1), a liver protein crucial for cholesterol homeostasis (219). It is responsible for reverse cholesterol transport by increasing the clearance of HDL by liver cells (220). Several studies have also reported a side effect of its use: pruritus as well limiting its use (218).
- Cilofexor: Non-steroidal molecule that reduces steatosis, downstages hepatic fibrosis (221).
- Tropifexor: Novel and highly potent agonist of FXR and is being used in stage 2 human clinical trials in patients (222).
- Vonafexor: A non-steroidal FXR agonist that has an action of reduction of liver fat content, fibrosis biomarkers, body weight and improving kidney function (223).

#### Using SGLT inhibitors and incretin-based approaches

Incretin-based therapies and sodium-glucose cotransporter 2 (SGLT-2) inhibitors are now being worked on as novel classes of glucose-lowering drugs used in the management of T2DM and are proving to be playing a simultaneous role in improving liver health (224) (Table 3).

- SGLT-2 inhibitors are antihyperglycemic agents that target SGLT-2 proteins expressed in the proximal convoluted tubules of the kidneys, where they normally mediate the reabsorption of glucose from the urine; by inhibiting this process, these agents promote increased urinary glucose excretion and help lower blood glucose levels (225).
- These drugs exert their effect by preventing the reabsorption of filtered glucose from the tubular lumen.
- Their beneficial effects range from weight loss, regulation of stress in the endoplasmic reticulum, oxidative stress, low-grade inflammation, apoptosis and autophagy (226).
- Incretins are hormones derived from the intestinal mucosa that play a key role in regulation of blood sugar levels as they stimulate secretion of insulin from pancreas post glucose intake (227).

**Table 3:** Antidiabetic drugs with potential hepatic benefits.

**Drug class**	**Name of drug**	**Mechanism of action and effect**
SGLT-2 inhibitors	Dapagliflozin	Reduced expression of hepatic inflammatory cytokines (TNF-α, IL-1β, IL-18) and improved hepatic steatosis; decrease in body weight as well (324).
	Empagliflozin	Improvement in hepatic inflammation and steatosis as seen in downregulation of inflammatory markers (325, 326).
Incretins	Glucagon-like peptide-1 (GLP-1)	Released from L-cells of the intestinal in response to nutrient ingestion and enhances glucose-stimulated insulin secretion; GLP-1 agonists & DPP-4 (enzyme dipeptidyl peptidase-4 that degrades GLP-1) inhibitors are of great interest for reduction of liver fat. GLP-1 agonists have shown action in weight loss as well as suppression of appetite and improvement in sensitivity of insulin. Examples are liraglutide and exenatide (327).
	Glucose-dependent insulinotropic polypeptide (GIP)	These are released from K-cells of the small intestine in response to glucose or fat ingestion, and potentiates glucose-stimulated insulin secretion (327, 328).

#### Role of AMP-activated protein kinase (AMPK)

AMPK activators are now gathering attention as alternatives to conventional treatments (228). These are involved in modulating energy metabolism under conditions of increased AMP:ATP ratio during energy deprivation. It further inhibits DNL gene expression by suppressing the actions of the enzyme acetyl-CoA carboxylase 1 pathway and promotes lipolysis through activation of the carnitine palmitoyl transferase 1 pathway in the liver.

Various agents that function as AMPK activators can be classified broadly into two categories:

A. Direct activators: Examples such as: A-769662 (229), Salicylate [both implied for improving liver function and reduction of hepatic fat].
B. Agents that mimics AMPK's downstream activity: Such as metformin (230) and aramchol (231) [reduction of hepatic fat content and inhibition of fatty acid synthesis in liver respectively].

AMPK is a critical regulator of cellular energy metabolism and oxidative stress defense. It has been identified as a central protein capable of mitigating cytotoxicity, suppressing inflammation, and preventing fibrosis. Hence, it has been positioned as a promising therapeutic target for addressing the primary drivers of MASLD.

### Targeting liver health

Currently, most of the effective therapeutic methods are targeting liver health. MASLD can't only be thought of as a precursor to T2DM, as liver damage has been shown to exacerbate diabetes by causing insulin resistance and beta cell failure. There are several molecular pathways that are thought to be involved in the process, and these are potential targets for therapeutic intervention. First, hepatocyte lipid overload and the presence of fat metabolites have been shown to activate serine kinase cascades that cause insulin resistance (44, 62, 232–235). So, blockade of these kinases may prevent the progression from MASLD to T2DM. There is an ongoing trial to assess the effectiveness of pioglitazone in treating advanced liver disease due to its insulin sensitizing effects, while lifestyle modification is a rational and safe therapy for MASLD with T2DM (236, 237). However, diabetes is typically characterized by compromised antioxidant capacity and increased oxidative stress. In patients with concurrent MASLD and diabetes, this oxidative imbalance exacerbates hepatic inflammation and fibrogenesis, worsening disease prognosis and dramatically increasing the risk of hepatocellular carcinoma and other liver diseases, while also elevating the risk of microvascular and macrovascular diabetic complications (238–244). High-dose vitamin E therapy has been shown to improve all aspects of liver histology in adults with MASLD (236, 245). It has also been shown to prevent the onset of T2DM in adults with metabolic syndrome and/or diabetes. High-dose statin therapy has been suggested as a treatment of MASLD. Triglyceride-lowering effects and improvement of aminotransferase levels were seen in early studies. However, recent evidence suggests that there is a risk of further liver damage (246, 247). These drug therapies are probably inappropriate for mild liver disease in T2DM and will have to be balanced with the risks and benefits. Ultimately, drug therapy for the liver in T2DM must be tailored to the individual.

### Lifestyle interventions and diabetes management

Lifestyle variables like excessive consumption of foods high in calories and a decrease in physical activity and exercise, are closely linked to the development of MASLD. Despite many negative effects as stated earlier, currently there are no pharmacological treatments for MASLD. Therefore, healthy lifestyle is the most important management of MASLD which involves diet, exercise and weight loss (248). Healthy lifestyle for both adults and children include eating a diet high in fruits, nuts, seeds, whole grains, fish, poultry along with regular physical exercise and avoiding excessive intake of red meat, ultra-processed foods, sugar-sweetened beverages, and meals fried at high temperatures (248). Several randomized controlled trials have shown lifestyle interventions reduce body weight, improve hepatic triglyceride content and improve MASLD activity score in patients suffering from MASLD (249–254). Furthermore, most studies show that changes in lifestyle are associated with improvements in cardiovascular disease (CVD) risk variables, including insulin resistance and blood cholesterol levels. Several clinical practice guidelines promote weight loss through calorie restriction as the best evidence-based strategy to improve MASLD across the disease spectrum (250, 252, 255). Comprehensive lifestyle modification should include dietary change to lower calorie intake, lifestyle and behavioral training, and increase physical activity. Avoiding smoking should also be part of the changes, as it has been linked to MASLD, fibrosis progression and HCC (256). Although several hypo energetic diets can reduce liver fat and promote weight reduction, the Mediterranean diet (MD) offers additional cardiometabolic benefits related to CVD risk reduction, which is the leading cause of mortality in most people with MASLD (257). However, their real-world feasibility across diverse, resource-constrained populations hinges on overcoming significant implementation barriers.

A mixed-methods trial in Northern England involving 19 MASLD patients demonstrated that after 12 weeks of MD counseling using meal plans and recipes, adherence increased from moderate to high, yielding modest weight loss (~2.4 kg) and HDL improvements. However, significant obstacles emerged, including an obesogenic environment, everyday stress, demand for convenience foods, and limited understanding of MASLD's health implications factors that adversely affected commitment to dietary changes (258–260). Similarly, a qualitative study in Australia with multicultural participants revealed that while the MD was perceived as more enjoyable and sustainable than a low-fat diet, barriers included access to culturally appropriate foods and sustaining changes post-interventions (260). A Tunisian study found patients could adhere to MD principles when fresh ingredients were affordable, and recipes were culturally tailored. Tunisian NAFLD patients had low MD adherence due to financial constraints and dietary adaptation challenges (261).

Systematic reviews highlight recurrent themes affecting MD adoption beyond Mediterranean regions: economic constraints, such as higher costs of fresh produce and olive oil; limited availability in local markets; cultural mismatches, and low nutrition literacy hindering behavior change. In regions with low socioeconomic status, MD adherence is strongly associated with greater food costs and younger age, while access to affordable, healthy food options especially in food deserts poses practical limitations (259, 262, 263).

Incorporating physical activity and exercise with dietary changes should be emphasized in the treatment of MASLD. Numerous randomized controlled trials have shown that exercise alone lowers liver fat in people with MASLD (264), whereas inadequate physical activity is linked to an increased risk for MASLD progression (265). Additionally, several recent studies have shown that a higher level of physical activity is linked to a lower risk of cirrhosis, liver fibrosis, and all-cause mortality (266–268). Exercise regardless of weight loss has hepatic and cardiometabolic benefits and it should be regularly advised and customized to patients' physical capabilities and preferences (269). Individuals with sedentary lifestyle and no physical activity should set achievable goals minimum of at least 150 min per week (30 min per day on 5 days per week) of moderate activity that includes anything that will raise the heart best and break a sweat still allowing to talk (269). According to current recommendations, a mix of resistance (also known as “strength,” like weight-lifting) and aerobic (often known as “cardio,” like brisk walking, cycling, and swimming) exercise should be employed (265, 269). Dietary and lifestyle changes must be adopted for life to prevent the progression of MASLD and its common comorbidities—namely, CVD and type 2 diabetes.

Treatment approaches for MASLD in diabetic patients primarily focus on improving insulin control and reducing liver fat accumulation. Achieving better glycemic control, as indicated by lower HbA1c levels, has been associated with reductions in ALT levels, which serve as a marker of liver inflammation (270, 271). For T2DM, effective management of hyperglycemia and improved insulin sensitivity are critical for lowering rates of DNL and reducing hepatic fat. These changes are difficult to achieve in practice and there are no current treatments that are highly effective. One promising therapeutic avenue involves enhancing the activity of peroxisome proliferator-activated receptor gamma (PPARγ). Thiazolidinediones (TZDs), which target this receptor, have shown potential in improving insulin sensitivity and reducing ALT levels in MASH patients (272, 273). However, due to their cardiovascular side effects, TZDs are not recommended for MASLD treatment (274). Additionally, anabolic hormone therapy to correct deficiencies or counteract the muscle wasting associated with MASLD/MASH represents a compelling area for future research.

Low glycemic index diets rich in monounsaturated fatty acids are associated with improvement in hepatic steatosis and ALT levels, and in a recent study, *ad libitum* low carbohydrate diets resulted in greater weight loss and improvement in insulin resistance compared to energy-restricted high carbohydrate diets (275–277). Weight loss has been associated with improvements in liver enzymes and histology in a number of different patient populations, and the associated metabolic improvements are likely to be mediated via reduction in adipose tissue inflammation and secretion of pro-inflammatory adipokines (278–281).

Animal studies have shown that a combination of exercise and dietary modification results in an alteration of the intestinal microbiota, an increase in the production of intestinal mucins, and a reduction of endotoxin and inflammatory cytokine production, which in turn prevents the development of steatosis (74, 143, 282–285). In human studies, increased physical activity has been associated with a reduced prevalence of hepatic steatosis, and a recent randomized control trial has shown that 12 months of moderate-intensity exercise in patients with T2DM resulted in reduced hepatic triglyceride content (286–289). Lifestyle interventions such as physical activity, dietary modification, and weight loss are the first line of therapy for the management of T2DM and are the most effective interventions for prevention of diabetes in high-risk populations (279, 290–292). Evidence is now emerging to suggest that these interventions may be effective in the prevention and management of MASLD and, in doing so, may influence the intestinal microbiota (286, 293, 294).

Altogether, these studies suggest that the beneficial effects of lifestyle interventions on both diabetes and MASLD could be mediated via modulation of the gut microbiota, and as our understanding of the mechanisms involved increases, it may be possible to make targeted therapeutic recommendations (154).

## Can conclusions drawn from animal studies sufficiently support translation to human pathophysiology?

While animal models especially mouse such as diet induced, deficiency induced, toxin induced, genetically induced or as a mixture of these modalities have provided foundational insights into the mechanisms of MASLD and MASH, the translation of these findings to human pathophysiology remains limited due to significant biological and metabolic disparities between species (295, 296).

The ob/ob and db/db models, for instance, exhibit key features of human metabolic syndrome and develop hepatic steatosis on a standard diet. When exposed to a secondary insult (e.g., methionine- and choline-deficient [MCD] diet), these models can progress to MASH like phenotypes. However, their translational relevance is restricted by the fact that congenital leptin deficiency (as seen in ob/ob mice) or leptin resistance (db/db mice) is extremely rare in humans (297–299). This limits their capacity to represent the etiology of human obesity, insulin resistance, and hepatic steatosis.

Moreover, the MCD diet model, while useful in inducing liver injury and inflammation, leads to metabolic alterations such as weight loss and decreased insulin levels that contrast starkly with human MASH, which is typically associated with obesity and insulin resistance. This explains the fact that many animal models either replicate histopathological features or metabolic features but rarely both (296).

Significantly, human clinical data have highlighted the limitations of these models. A study utilizing a human liver chimeric mouse model revealed striking differences in molecular responses between murine and human hepatocytes when exposed to a Western diet, indicating species-specific liver functions and responses (300). Another study revealed there was partial overlap in liver transcriptome profiles between mice and humans, the gene expression patterns in mouse models remained distinctly different from those in humans, indicating that the pathophysiology in mice does not fully replicate human MASLD (301). In another study Vacca et al., did a retrospective study and assessed mouse models using a human proximity score derived from metabolic phenotypes, liver histopathology and transcriptomic similarity to human liver data (302). They concluded from the study that Western style diets especially those with added cholesterol and longer feeding are the closest match across metabolic, histological, and molecular layers and Choline-deficient or genetically driven models may help elucidate specific fibrosis mechanisms but do not recapitulate the full human disease spectrum (302).

Thus, animal models remain invaluable for understanding discrete mechanisms or stages of MASLD/MASH. However, no single mouse model accurately recapitulates the integrated metabolic, histological, and molecular complexity of human disease. While existing models continue to guide mechanistic research and therapeutic testing, further development of models that more closely align with human pathophysiology is crucial to improve translational validity (295, 303, 304).

## Conclusions and perspectives

The multifaceted nature of MASLD emerges through its complex interactions with IR and T2DM, with the gut microbiome serving as a central orchestrator of disease progression. Current cross-sectional studies inadequately address fundamental relationships between gut dysbiosis and hepatic dysfunction, demanding comprehensive longitudinal microbiome investigations with standardized protocols to delineate temporal disease progression patterns. Rigorous randomized controlled trials evaluating microbiome-targeted therapies, including FMT and precision probiotics, are essential for clinical translation of emerging mechanistic insights. The complex host-microbe-metabolic interactions underlying MASLD pathophysiology require sophisticated multi-omics integration strategies, employing advanced computational approaches to identify novel biomarkers and therapeutic targets. These complementary research strategies will accelerate the development of precision diagnostics and mechanism-based interventions, transforming MASLD management from reactive treatment to proactive, personalized medicine approaches. Given projected increases in global MASLD prevalence, this integrated research framework represents our most promising pathway toward effective therapeutic solutions for this increasingly prevalent metabolic liver disorder.
